# Field-Scale Testing of a High-Efficiency Membrane Reactor (MR)—Adsorptive Reactor (AR) Process for H_2_ Generation and Pre-Combustion CO_2_ Capture

**DOI:** 10.3390/membranes14020051

**Published:** 2024-02-11

**Authors:** Nicholas Margull, Doug Parsley, Ibubeleye Somiari, Linghao Zhao, Mingyuan Cao, Dimitrios Koumoulis, Paul K. T. Liu, Vasilios I. Manousiouthakis, Theodore T. Tsotsis

**Affiliations:** 1 Chemical and Biomolecular Engineering Department, University of California, Los Angeles, CA 90095, USA; nicholas@margull.net (N.M.); isomiari@ucla.edu (I.S.); vasilios@ucla.edu (V.I.M.); 2 Media and Process Technology, Inc., Pittsburgh, PA 15328, USA; dparsley@mediaandprocess.com (D.P.); pliu@mediaandprocess.com (P.K.T.L.); 3 Mork Family Department of Chemical Engineering and Materials Science, University of Southern California, University Park, Los Angeles, CA 90089, USA; linghaoz@usc.edu (L.Z.); caomingy@usc.edu (M.C.); 4 Institute for Decarbonization and Energy Advancement, University of Kentucky, Lexington, KY 40507, USA; dimitrios.koumoulis@uky.edu

**Keywords:** membrane reactor (MR), adsorptive reactor (AR), H_2_ generation, CO_2_ capture, carbon molecular sieve membrane

## Abstract

The study objective was to field-validate the technical feasibility of a membrane- and adsorption-enhanced water gas shift reaction process employing a carbon molecular sieve membrane (CMSM)-based membrane reactor (MR) followed by an adsorptive reactor (AR) for pre-combustion CO_2_ capture. The project was carried out in two different phases. In Phase I, the field-scale experimental MR-AR system was designed and constructed, the membranes, and adsorbents were prepared, and the unit was tested with simulated syngas to validate functionality. In Phase II, the unit was installed at the test site, field-tested using real syngas, and a technoeconomic analysis (TEA) of the technology was completed. All project milestones were met. Specifically, (i) high-performance CMSMs were prepared meeting the target H_2_ permeance (>1 m^3^/(m^2^.hbar) and H_2_/CO selectivity of >80 at temperatures of up to 300 °C and pressures of up to 25 bar with a <10% performance decline over the testing period; (ii) pelletized adsorbents were prepared for use in relevant conditions (250 °C < T < 450 °C, pressures up to 25 bar) with a working capacity of >2.5 wt.% and an attrition rate of <0.2; (iii) TEA showed that the MR-AR technology met the CO_2_ capture goals of 95% CO_2_ purity at a cost of electricity (COE) 30% less than baseline approaches.

## 1. Introduction

Integrated Gasification Combined Cycle (IGCC) power plants with carbon capture and storage (CCS) promise to produce electricity from fossil fuels with no harmful CO_2_ emissions [1,2,3,4,5,6]. In these plants, the fossil fuel (e.g., coal or biomass), rather than being combusted directly to produce power, is instead converted in a gasifier [7,8] into syngas, a gas mixture containing H_2_/H_2_O/CO_2_/CO (and N_2_ when an air-blown gasifier is used). This syngas is cooled down and treated in a cold-gas clean-up unit (CGCU) to remove trace contaminants (e.g., H_2_S, COS, NH_3_) and is then heated up to the desired temperature to react with steam, via the water gas shift (WGS) reaction (Equation (1)), to convert the

CO + H_2_O ↔ CO_2_ + H_2_ (1)

CO into H_2_ and CO_2_ [8,9] in a cascade of high-temperature shift (HTS) and low-temperature shift (LTS) reactors [10,11]. For CCS, the CO_2_ in the reactor outlet is removed in absorption columns [7,12], and after drying and pressurization, is directed to permanent underground storage [11] or finds beneficial uses (e.g., enhanced oil recovery (EOR)). The H_2_ is mixed with N_2_ as a diluent and sent to a turbine to generate electricity [7,13,14].

IGCC with CCS plants entail higher capital costs than plants without CCS due to their technical complexity involving multiple reaction, separation, and clean-up steps, and are perceived as economically prohibitive for near-term market deployment. In recent years, novel approaches have been explored to improve the technology, with reactive separation processes (such as membrane reactors (MRs) and adsorptive reactors (ARs)) being dominant [9] among them because they integrate reaction and separation steps in a single unit, which improves process efficiency [15,16,17,18,19], resulting in energy and cost savings [7]. 

MRs employing hydrogen-selective membranes have been used for H_2_ production from syngas [8,10,11] via the WGS route, with potential advantages that include lower operating temperatures and reduced steam, catalyst, and downstream purification requirements [8,9,10,11,20]. Pd and Pd-alloy [21,22,23,24], amorphous silica [2,8], and dense polymeric membranes [25,26] have all been utilized (see [2] for operating performance comparison) but they are not suitable, however, for use in harsh IGCC conditions [27,28]. Carbon molecular sieve membranes (CMSMs) are a promising alternative due to their robustness [8,29], and a CMSM-based WGS-MR was studied by this group [8] for contaminant-free H_2_ production in IGCC [30]. ARs employing a solid adsorbent for in situ CO_2_ removal also show promise for application to IGCC with CCS [2,6,13,31,32,33,34,35], with advantages that include lower operating temperatures and catalyst costs, increased safety [7], and decreased energy requirements for CCS by producing a pure CO_2_ stream ready for sequestration. The IGCC with CCS process dictates using the right sorbent that operates at high temperatures (523–573 K) and pressures (25 bar or higher) and has low cost, high CO_2_ adsorption capacity and selectivity, fast and stable sorption kinetics, and high thermal/chemical/mechanical stability [36]. Hydrotalcite-like materials are the sorbent of choice [5,8,33] as the sorption capacity of common adsorbents (e.g., zeolites, silica, MOFs, active carbons, etc.) rapidly declines with temperature. 

**Figure 1:**
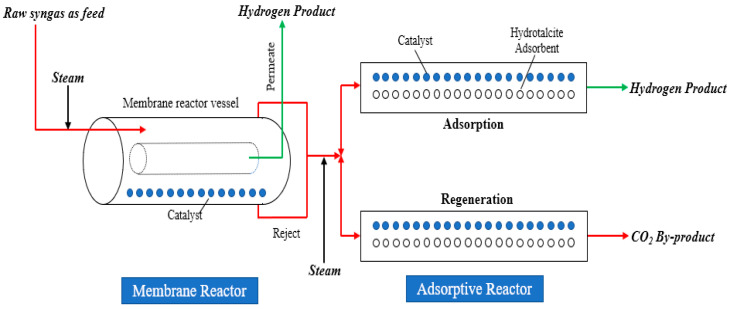
The schematic of the MR-AR Process.

A hybrid (named the HAMR) process that combines an AR and a MR in a single unit was proposed by this group [16], which, by simultaneously separating/removing both H_2_ and CO_2_, attained improved performance over standalone (MR or AR) systems. Several papers have since appeared using the process for the WGS reaction to obtain high-purity H_2_ with simultaneous CO_2_ capture [16,19,20,23,37,38,39,40]. Recently, this group proposed a new process (see Figure 1 for a schematic) for application to IGCC with CCS that offers enhanced conversion and separation performance by simultaneously removing both H_2_ and CO_2_ in situ.

But unlike the HAMR system, in the new process, the MR and AR units are physically separated, which offers added flexibility for the IGCC with CCS application by allowing one (i) to separately optimize the conditions for each individual unit without interference from the other (e.g., the CMSMs have an operating temperature threshold of ~350 °C, and co-locating the adsorbent and membrane in the same unit may adversely impact adsorbent regeneration); (ii) to operate the MR at steady state while the AR units are cycled between reaction and regeneration modes, which is an important advantage as it lessens the burden on membrane seals and materials during operation. This integrated MR-AR system can simultaneously maximize CO conversion, H_2_/CO_2_ recovery, and purity and deliver a high-pressure pure CO_2_ stream [7,13], and shows significant advantages over the standalone MR and AR systems, which is the result of the added synergy created from the two individual units brought together [15].

In this paper, the findings of a field-scale study are presented, aiming to validate the technical feasibility of this integrated MR-AR process with real syngas. Prior to the initiation of this effort, the process was tested in the laboratory on simulated syngas and important performance attributes and requirements were established. Specifically, this team’s past efforts preceding the initiation of this field-scale study involved the following key developments and Tasks (for further details about the laboratory study, see recent publications by the team [7,13]):

- The lab-scale experimental MR-AR system was designed, constructed, and tested, appropriate CMSM, adsorbents, and catalysts were selected and characterized, and experimentally validated relevant multi-scale mathematical models were developed. Subsequently, the proposed process was experimentally tested using a simulated gasifier off-gas (from both air-blown and oxygen-blown coal gasifiers), and based on the lab-scale results, an initial technical and economic feasibility study was completed.
- For use in the lab-scale experiments, “state-of-the-art” leading CMSMs we prepared with exceptional performance meeting all the original project targets (set forth by US DOE, which funded the laboratory study): H_2_ permeance (1 to 1.5 m^3^/m^2^/h/bar, or 370.3 to 555.5 GPU) and a H_2_/CO selectivity of >80 in the relevant temperature (up to 300 °C) and pressure conditions (up to 25 bar). The CMSMs exhibited very robust and stable performances during a continuous long-term run (over >500 h of H_2_S exposure at 25 bar of pressure) and maintained high He/N_2_ (~126) and H_2_/CO (~100) selectivities over a total of 742 h of H_2_S exposure. The same type of CMSM but with a larger length was utilized in the pilot-scale project.
- Hydrotalcite (HTC) adsorbents were prepared and characterized in high (up to 30 bar) pressure conditions. These materials showed maximum CO_2_ uptake capacities of >10 wt.% and working capacities under cyclic AR conditions of ~3 wt.% and exhibited stable performance during CO_2_ cycling in various atmospheres, including a >500-h continuous MR-AR run. A commercial sour-shift catalyst was utilized in the lab-scale experiments, and data-validated global rate expressions were developed to simulate the lab-scale MR-AR system as well as in the generation of a preliminary process technoeconomic analysis (TEA). The same adsorbents and catalysts were used in the field-scale project.
- The integrated MR-AR lab-scale system was tested during numerous multiple-cycle runs with simulated gasifier off-gas and displayed superior performance to that of a conventional packed bed reactor (PBR) generating a high-purity H_2_ product, which is directly usable in a hydrogen turbine for power generation. A key conclusion from the lab-scale study that motivated the field-scale efforts was that the CMSM, catalyst, and adsorbent were very robust and stable under the large H_2_S concentration, high-temperature, and high-pressure IGCC-like environment during the long-period lab-scale MR-AR multiple-cycle run (similar in duration to the field-scale test).

The field-scale project has advanced the MR-AR technology by testing a 100× scaled-up analog of the lab-scale MR-AR system with an actual coal gasifier off-gas. It was carried out in two different phases, separated by an intervening ”go-no-go” review step. In Phase I of the project, the team designed, constructed, and assembled the field-scale MR-AR system, prepared the CMSM, adsorbents, and catalysts, tested the unit with simulated syngas to validate functionality, and prepared a preliminary TEA of the technology. In Phase II, the team installed the unit at the test site (the CAER Center at the University of Kentucky (UKy)) and completed all utility connections and hook-ups to the gasifier, field-tested the MR-AR process using real coal gasifier off-gas, collected and analyzed experimental data, and completed a detailed TEA of the technology. 

All project milestones and success criteria set forth by the US DOE (that also supported the field-scale study) were met. Specifically, the team (i) prepared high-performance CMSM tubes (ID: 3.5 mm, OD: 5.7 mm, 76.2 cm long) that meet the target H_2_ permeance (>1 m^3^/(m^2^.h.bar) or (>370.3 GPU)) and a target H_2_/CO selectivity of >80 in the relevant temperature (up to 300 °C) and pressure conditions (up to 25 bar) with a <10% decline in performance over each 250 h testing period; (ii) procured a commercial sour-shift catalyst in sufficient quantity and prepared up to 10 kg of the pelletized adsorbent for use in relevant conditions (250 °C < T < 450 °C, pressures up to 25 bar) with a target adsorbent working capacity of >2.5wt.% and a target sorbent attrition rate of <0.2; (iii) installed the field-scale unit at the test site (UKy) and tested the MR-AR process using real coal gasifier off-gas for over 500 h in both static and flow experiments; (iv) updated the TEA from the lab-scale study based on field-scale data and met CO_2_ capture goals of 95% CO_2_ purity at a cost of electricity 30% less than baseline capture approaches. Key technical results from the study are presented in this paper. Additional information, including more complete details about materials, methods, and procedures can be found elsewhere [41]. 

## 2. Results and Discussion

### 2.1. Design, Construction, Assembly, and Preliminary Testing of the Field-Scale Unit 

A field-scale experimental MR-AR system that was a scaled-up (100×) analog of the lab-scale system was designed, constructed, and assembled. This effort began with the design of the system, which was based on in-house models developed during the lab-scale study and further upgraded during the present study for use in the design phase. The various pieces of equipment necessary to construct/assemble the unit were either procured from various external vendors or fabricated by the project team. 

**Figure 2:**
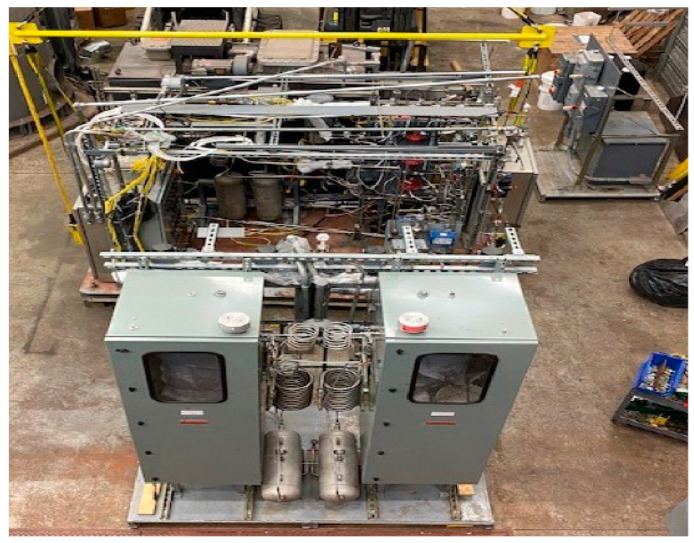
Overhead view of pilot-scale unit installed at CAER.

The field-scale system consisted of (i) the gas delivery system (for connecting the unit to the gasifier); (ii) the MR containing the CMSM and the WGS catalyst (see Figure S1 in the Supplementary Materials section) housed in an explosion-proof oven; (iii) the AR subsystem containing the adsorbent and catalyst with its appropriate valves and control hardware (see Figure S2 in the Supplementary Materials section); (iv) the overall system control hardware incorporating various system automation and safety features to minimize the human-error potential and to facilitate extended long-term continuous operation and data acquisition; and (v) the analysis section equipped with the appropriate analytical equipment. A photograph of the pilot-scale unit, as installed at CAER at the UKy, is shown in Figure 2. 

The AR system consisted of two units (equipped with individual explosion-proof heaters) for continuous cycling between adsorption/reaction and desorption/regeneration, each consisting of three reactors in series (see Figure S2). The decision to employ two ARs in parallel was taken based on project cost considerations and for simplicity of operation. For continuous operation of two banks of three ARs in series, with one bank of reactors regenerating and the other adsorbing at any given moment, equal adsorption and regeneration times are, however, necessary. Given that desorption is slower than adsorption, selecting the switching time entails process optimization. 

This is because selecting a short switching time (not sufficiently long for complete regeneration of the adsorbent bed) means that subsequent adsorption cycles will not start with a fresh adsorbent bed. Selecting a long time, on the other hand, past adsorbent saturation during the adsorption/reaction part of the cycle, will mean that the AR will operate sub-optimally (i.e., it will function as conventional PBR) for a good fraction of time during that part of the cycle. 

**Figure 3:**
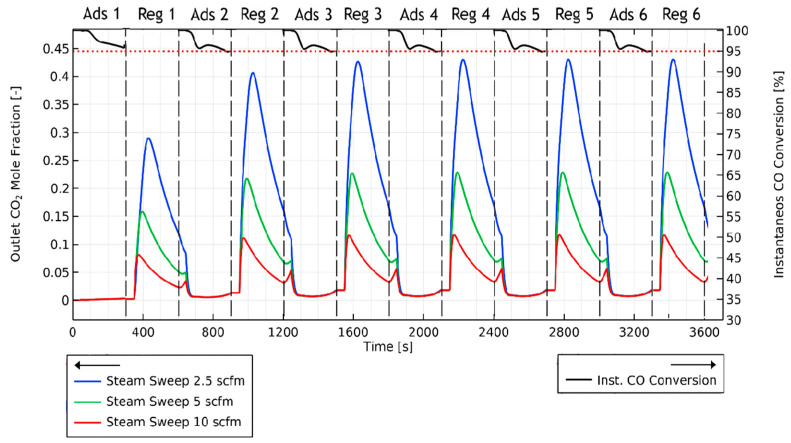
Outlet CO_2_ mole fraction for different regeneration steam sweep rates (left vertical axis) and instantaneous CO conversion during adsorption phases (right vertical axis) at the AR outlet.

Figure 3 shows the results from such a simulation carried out during the design phase of the field-scale unit for conditions relevant to the planned operation of the unit at the field site (for the AR model used in these simulations, see [42], and for its experimental validation, see further discussion in Section 2.2 below). The switching time for this run was selected to be 5 min (based on an initial single adsorption run for the AR loaded with fresh adsorbent that revealed a CO_2_ breakthrough time of ~6 min), while the other parameters for the simulation can be found in Table S1 in the Supplementary Materials section). The cycle simulations begin with initial adsorption in a bed of fresh adsorbent (AR filled with steam at 523 K), followed by subsequent regeneration and adsorption cycles. It is found that within a total of six such adsorption/regeneration cycles, long-term stable behavior is attained. Figure 3 shows the outlet CO_2_ mole fraction (left vertical axis), as well as the instantaneous CO conversion (right vertical axis) during an adsorption/reaction six-cycle run.

For use in the field-scale tests, CMSM tubes, 30″ (76.2 cm) long, were prepared at M&PT and potted into 2″ (5.08 cm) outside diameter (OD) membrane bundles (containing between 7 to 18 membrane tubes each). The individual membrane tubes (and the resulting bundles) were quality-tested at M&PT under non-reactive conditions for their separation characteristics, routinely with inert gases, i.e., He and N_2_, and for a select number of such membranes with a range of other test gases as well (e.g., He, H_2_, CO, N_2_, and/or CO_2_). 

Sufficient quantities of adsorbent pellets (made from powders of a Mg-Al layer double hydroxide material, prepared by a procedure previously developed by this team [43]) were also prepared for the pilot-scale test. The pelletization technique was developed by testing various binder formulations and different pressing techniques of the resulting hydrotalcite/binder mixtures. Preliminary screening among the various techniques was based on the testing of physical properties, i.e., particle density and porosity, axial and radial strength, and overall physical appearance. For the techniques that successfully passed the initial screening, continued development focused on the adsorbent’s physical robustness during thermal cycling and on the scaling-up of the preparation procedure to transition from making small sorbent batches for laboratory testing to being able to prepare the large quantity of adsorbents needed for the field testing (for further details about the materials used and the pelletization methods employed, please see [41]).

**Figure 4:**
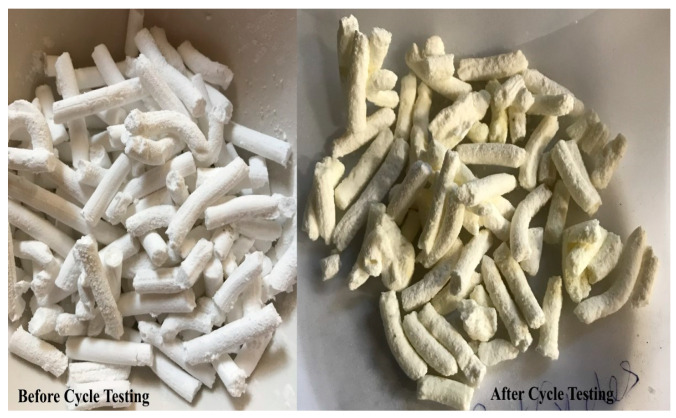
Photographs of the adsorbent pellets before and after the cyclic testing.

A photograph of a number of adsorbent pellets (Batch #33), prepared by the final pelletization technique selected, is shown in Figure 4 (left). In the same figure (right), a photograph is also included of the same adsorbent pellets after they had undergone numerous adsorption/regeneration testing cycles, under pressure/temperature conditions relevant to the field testing for a total of 1000 h of experimentation. A post-cycle-testing analysis of the pellets showed minimal damage and erosion to the pellets and no apparent change in mechanical strength.

**Figure 5:**
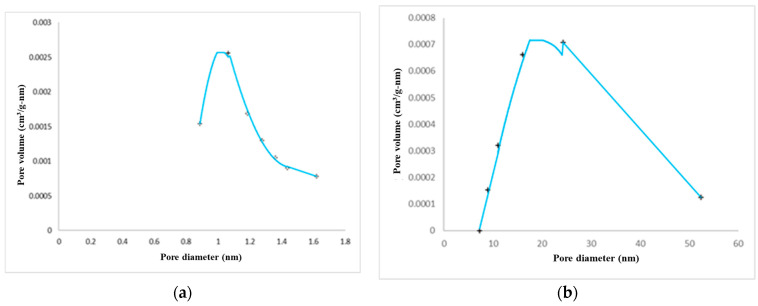
Pore size distribution of Batch #33 pellets: (**a**) Horvath–Kawazoe differential pore volume; (**b**) BJH adsorption dV/dD pore volume.

Pellets from Batch#33 were analyzed using the BET technique to measure their surface area, pore volume, and pore size distribution (PSD), both intact and in their powder form (after grinding). Figure 5 shows the measured PSD, which is bimodal consisting of mesopores with an average pore diameter of ~23 nm and a microporous region with an average pore diameter of ~0.9 nm. The powder and the pellet materials have pore structural properties that are similar to each other. A total of 10 Kg of Batch#33 adsorbent pellets were prepared for the field testing. 

Two different commercial Co/Mo/Al_2_O_3_ sour-shift catalysts (catalysts #1 and #2) were available for the project. Catalyst #1 was a newer batch of the catalyst that was employed in the predecessor lab-scale project. Catalyst #2 was employed by M&PT in a previous field-testing project at CAER. The kinetics of both types of catalysts were investigated because they were not known for catalyst #2, and for catalyst #1, it was desired to make sure that the reaction kinetics developed in the lab-scale project were applicable to the new catalyst batch purchased. Knowing these kinetics was also important in terms of developing a global rate expression to be used in the multi-scale MR and AR models for equipment design and scale-up.

The experiments were carried out in the high-pressure and temperature lab-scale MR-AR system at USC operating as a PBR. For the reaction kinetics studies, the reactor operated under isothermal and isobaric conditions. A 1-D PBR model was utilized to analyze the experimental data of both catalysts (for further details, see [8]). The applicability of three different microkinetic rate models, previously developed for a commercial Co/Mo catalyst by Osa et al. [33,34]) and tested for catalyst #1 in the previous project, was tested with global reaction rate expressions as follows:

Formate intermediate model:

(2) $$r=\frac{k_{w}(P_{CO}P_{H_{2}O\;}-\frac{P_{CO_{2}}P_{H_{2}}}{K_{eq}})}{{\left(1+K_{1}P_{CO}+K_{3}P_{H_{2}O}\right)}^{2}}$$

Associative mechanism:

(3) $$r=\frac{k_{w}(\frac{P_{CO}P_{H_{2}O\;}}{{P_{H_{2}}}^{\frac{1}{2}}}-\frac{P_{CO_{2}}{P_{H_{2}}}^{1/2}}{K_{eq}})}{{\left(1+K_{1}P_{CO}+K_{3}\frac{P_{H_{2}O}}{{P_{H_{2}}}^{1/2}}\right)}^{2}}$$

Direct oxidation (DO) mechanism:

(4) $$r=\frac{k_{w}(P_{CO}P_{H_{2}O\;}-\frac{P_{CO_{2}}P_{H_{2}}}{K_{eq}})}{1+\frac{KP_{CO}P_{H_{2}O}}{P_{H_{2}}}}$$

**Figure 6:**
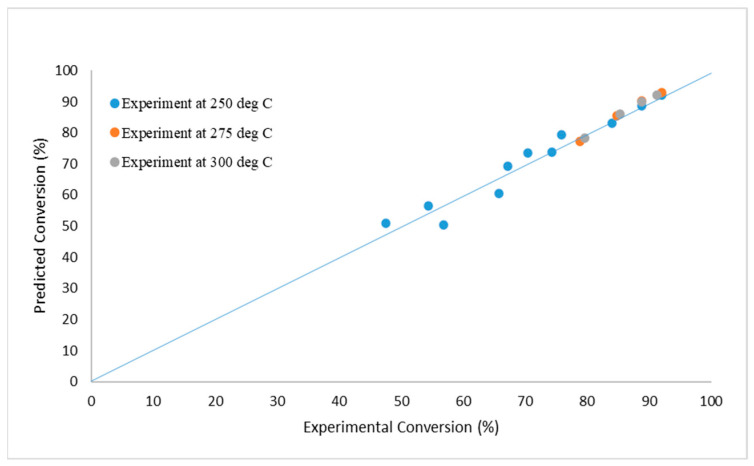
Kinetics data for catalyst #2, and parameter fit using the Associative mechanism.

For catalyst #2, the Associative mechanism described the experimental data the best (see Figure 6). For catalyst #1, as with the lab-scale data, among the microkinetic models, the Direct Oxidation mechanism describes the experimental data the best.

In the lab-scale study, an empirical rate model was also shown to describe the kinetics for catalyst #1equally well (in fact, slightly better), as follows:

(5) $$r=kP_{CO}^{a}P_{H_{2}O}^{b}P_{{Co}_{2}}^{c}P_{H_{2}}^{d}(1-\beta )$$

where:

$$\beta =(\frac{1}{K_{eq)}}\cdot \frac{P_{CO_{2}}P_{H_{2}}}{P_{CO}P_{H_{2}O}})$$

Commercial catalyst #2 was shown in the reaction kinetics investigations to be more active than catalyst #1, but in the field-scale studies reported here that aimed to validate the functionality of the MR-AR process, catalyst #1 was selected. There were two reasons for such a choice: (i) a larger quantity of catalyst #1 was available in-house and (ii) the team had significantly more working experience with catalyst #1, having had thousands of hours of continuous operation during the lab-scale project. The global rate expression of Equation (5) was, therefore, also used in the MR and AR models for equipment design and scale-up.

**Figure 7:**
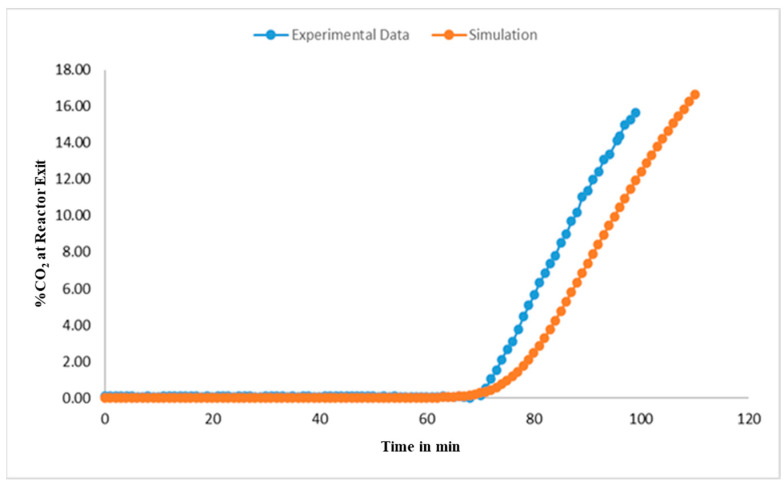
AR A adsorption experiment and simulation test results.

Prior to shipping the pilot-scale unit to UKy-CAER, AR adsorption test runs were carried out at the M&PT laboratories using simulated gas streams to assess adsorption performance. The experimental details for one of the AR adsorption test runs are shown in Table S2, and the corresponding experimental data and simulation results using the in-house models developed are shown in Figure 7. As can be seen in Figure 7, the experimental CO_2_ breakthrough time, defined as the time at which the CO_2_ molar fraction at the outlet reaches 1%, is 72 min in the specified operating conditions in Table S2. The simulation results are consistent with this CO_2_ breakthrough time, but they slightly underestimate the amount of CO_2_ leaving the saturated bed following the breakthrough time. Possible explanations for this discrepancy include uncertainties in the CO_2_ adsorption rate model and its parameters, as well as fluctuations in the experimental feed flow rate. Nevertheless, the key conclusion from these simulations is that the AR model provides a reasonable prediction of the experimental CO_2_ adsorption behavior of the bench-scale AR system, particularly in providing an accurate estimate of the breakthrough time, which is an important system operating parameter.

**Figure 8:**
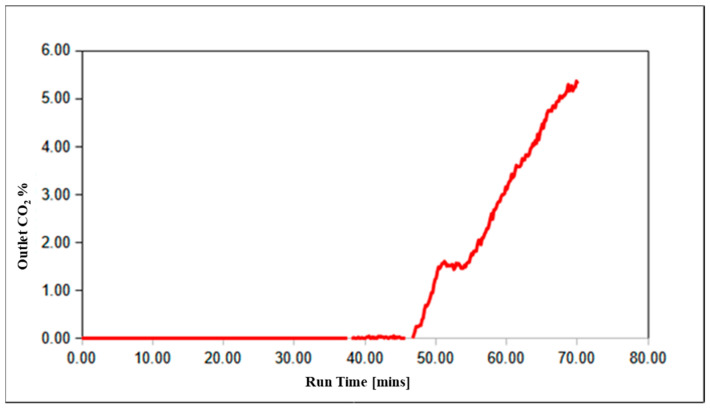
CO_2_ breakthrough data from AR A unit during a combined MR-AR run at M&PT.

Prior to shipping and installation at the test site, the field-scale MR-AR system underwent preliminary “shakedown” testing with simulated coal gasifier off-gas and other relevant gas mixtures to validate system functionality and operation and to gain operating experience. An example of such a test run is shown in Figure 8. 

For this run, the temperature of the MR oven was set to 255 °C, the external heaters for the ARs were set to 275 °C, the temperature of the MR steam generator was set to 245 °C, and the temperature of the steam generator for AR regeneration was set to 418 °C. A simulated gasifier syngas mixture with a composition of 44% H_2_, 7% N_2_, 23% CO, 25% CO_2_, and 1200 ppm H_2_S (simulating the oxygen-blown gasifier syngas at the UKy test site) was fed to the MR at a pressure of ~10 bar and flow rate of 0.5 scfm (14.16 lpm—for the results reported in Figure 8, the MR was run in its PBR configuration, i.e., with the permeate side exit being closed) together with steam at a flow rate of 4.33 slpm. The outlet gas from the MR served as the feed to the ARs for the testing of the MR-AR combined system. Before the start of the run, the reactors were brought to the operating temperature under a N_2_ purge. The MR was then switched over to running on syngas and steam and was allowed to stabilize for around two hours in order to reach a steady state before sending the gas from the reject side to the ARs. The AR outflow was monitored for its CO_2_ content with a real-time NDIR CO_2_ analyzer while the MR streams were monitored with a gas chromatograph, sampling every 10 min. Additional data points were taken periodically by the gas chromatograph to monitor other components of the AR outflow. AR breakthrough time was established as the time at which 1.0% or more CO_2_ was seen by the analyzer on the AR outlet. Figure 8 shows the CO_2_ breakthrough data for the AR unit A. For this run, initial breakthrough results indicated a working capacity of >>3 wt.%.

### 2.2. Installation and Operation of the Field-Scale Unit at the Test Site

Upon completion of the “shakedown” testing phase of the project, the MR-AR pilot-scale system was transported to the UKy-CAER test site and connected to the gasifier for the field-testing phase to commence with real gasifier syngas. Two different field-testing campaigns were conducted. In between these two campaigns, there was a “hiatus” period during which the experimental results from the first testing campaign were evaluated. During this “hiatus” period, any modifications needed for the field-scale system were implemented, and additional quantities of membranes and adsorbents were prepared for use during the second testing period. The data collected during both testing periods were used to quantify the CO conversion and H_2_/CO_2_ recovery and purity to provide the empirical information needed to complete the final process TEA (see further discussion below in this paper). A key focus of the field testing was the evaluation of the chemical/mechanical stability of membranes/adsorbents/catalysts following exposure to the various impurities encountered in real syngas. All three key materials proved robust to the field-testing environment and conditions.

The first field-testing campaign began with a series of MR-AR experiments intended to study, in a systematic fashion, the impact of key parameters for the various modes of AR adsorption and regeneration. In these tests, the syngas was introduced into the MR and allowed to run long enough for it to stabilize and generate representative gas concentrations in the reject and permeate streams. After that, the AR beds were switched on, one at a time, to receive the MR reject gas stream, with or without make-up steam. For all these preliminary runs, the ARs were allowed to run past the breakthrough point of 1% CO_2_ in the outflow stream, in some instances all the way to saturation, in order to generate baseline data on AR performance beyond the breakthrough level. For AR regeneration, a mixture of steam and N_2_ was employed (out of experimental expedience—in a commercial unit, only steam can be used for the regeneration step), but for some of the runs before initiating the flow of the steam/N_2_ gas mixture, in order to provide additional data for model validation, the bulk phase gas (“fill-gas”) in the AR bed was first bled-off with flowing N_2_. 

**Figure 9:**
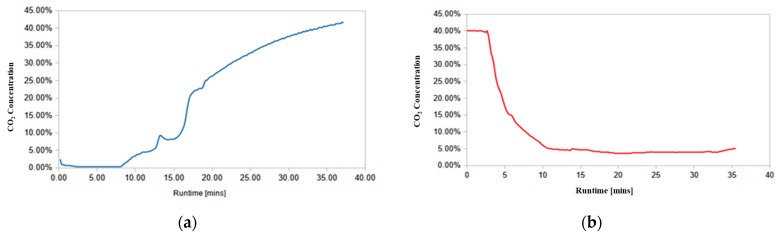
Experimental data for AR Run#4: (**a**) adsorption/reaction process; (**b**) regeneration process.

The quality of syngas at the testing site was quite variable, and for some of the runs involving poor-quality syngas, additional steam was added from the make-up steam system to the MR reject stream prior to being directed into the AR unit. The results from one of the runs are shown in Figure 9, which shows the CO_2_ concentration in the exit stream from one of the ARs (AR A) during the adsorption/reaction and regeneration parts of the experiment. For this experiment, the temperature of the MR oven was kept at ~254 °C and the pressure at 238 psig (17.4 bar). The syngas feed composition for the duration of the experiment was (H_2_ = 15.8%, N_2_ =25.3%, CO = 18.4%, and CO_2_ = 40.5%), its flow rate was 1.41 scfm (39.93 slpm), and the steam flow rate 0.89 scfm (25.20 slpm). During the adsorption/reaction part of the cycle, the reactor wall temperature (measured at the midpoint of the reactor) was set at 265 °C, the pressure was 254 psig (18.51 bar), and the MR reject stream was augmented with a steam flow of 1.52 scfm (43.04 slpm). During the regeneration, the reactor wall temperature was set to 265 °C, the pressure was 250 psig (18.25 bar), and the regeneration gas stream consisted of 0.25 scfm (7.08 slpm) N_2_ and 2.16 scfm (61.16) steam with an entering temperature of 356 °C. The breakthrough time for the adsorption/reaction part of the cycle (defined as the time when the concentration of CO_2_ in the exit stream reached 1%) was ~9.2 min.

**Figure 10:**
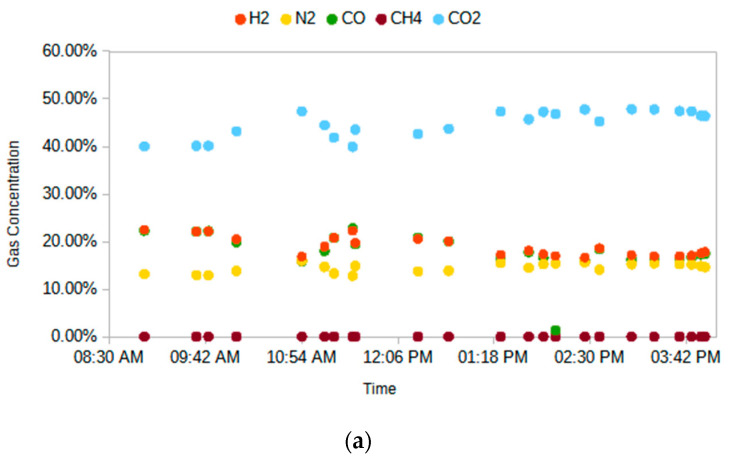

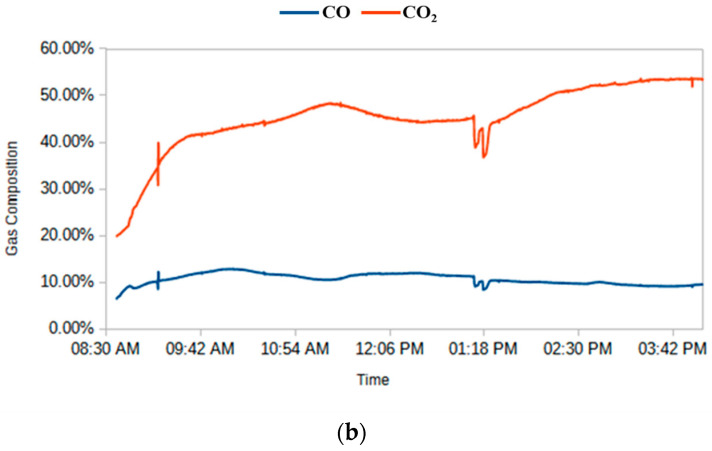
MR experiments: (**a**) syngas feed composition; (**b**) MR reject-side CO and CO_2_ composition.

A series of cyclic experiments were conducted with the goal of understanding the MR behavior and validating the kinetics and models developed during the lab-scale study and Phase I of this project. A key challenge here, in terms of being able to compare model results with experiments, was to identify conditions for which gasifier performance was stable and the syngas feed composition stayed relatively constant. Figure 10 shows the data from one of these experiments during a time period for which the syngas composition stayed relatively stable.

Table 1 compares the experimental MR CO conversion with the MR model predictions for the data shown in Figure 10. The experimental conversion data and the model predictions, as well as the operating conditions for three other experiments carried out on different dates during periods for which gasifier performance remained relatively stable, are also shown in Table 1. Comparing the measured and simulated MR CO conversions for all four datasets in Table 1 shows that the MR model does a good job of predicting the experimental MR CO conversions. The relatively small differences between the experimental values and the model conversion can be attributed to the uncertainty in determining the experimental values due to the variability of the feed compositions, as noted above.

**Figure 11:**
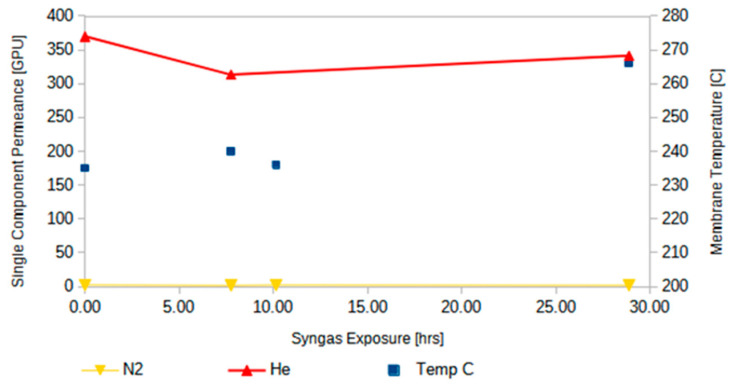
Stability evaluation of the CMS membrane bundle.

One of the key objectives for the testing of the MR component of the MR-AR system was validating membrane performance stability. During the MR experiments described above, when the opportunity arose (e.g., no syngas available from the gasifier), the membrane permeation properties were tested with single-gas permeation experiments with He (serving as surrogate gas for H_2_) and N_2_ (as surrogate gas for CO). The single-component membrane permeation data are shown in Figure 11, indicating no significant performance degradation after ~25 h of syngas exposure. Post-mortem tests have also been performed with several of the membrane modules utilized in the field study, which similarly showed no signs of performance degradation.

**Figure 12:**
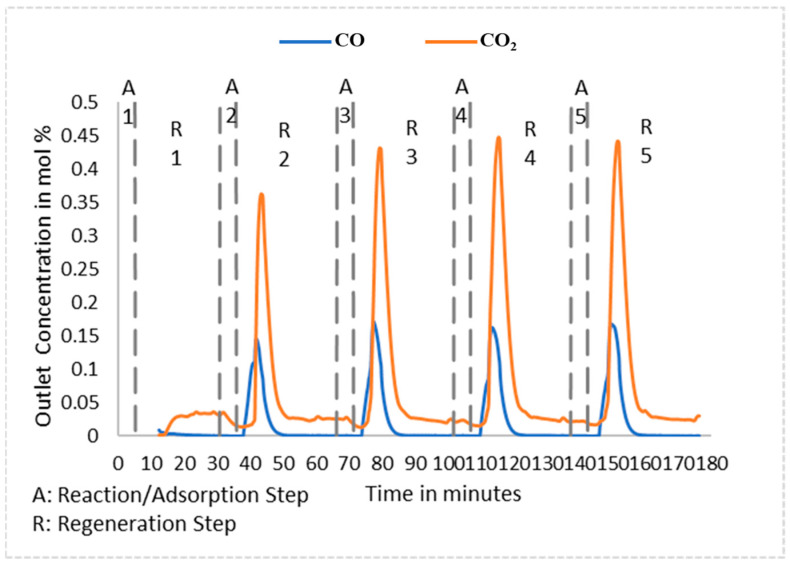
CO and CO_2_ concentration in the AR A exit stream showing cyclic behavior.

Several experimental runs were also carried out specifically focused on investigating AR performance during cyclic operation. In order to efficiently utilize the time that the gasifier was available to the team and exhibited stable performance, for these experiments, two of the three vessels in the AR A were disconnected from the series of reactors, with the remaining single-vessel AR A unit then being used for the cyclic behavior studies. To accommodate this change, the MR-AR system was re-configured for AR A to receive only a fraction (~30%) of the MR reject stream as feed for the adsorption–regeneration cyclic tests. For these cyclic tests, the adsorption run was carried out for ~8 min, which is less than the CO_2_ breakthrough time, after which the AR A was switched to regeneration mode for ~40–45 min. The exit CO and CO_2_ concentrations during a few consecutive cycles for one of the cyclic tests are shown in Figure 12.

For the cyclic run in Figure 12, the CO_2_ concentration reached periodic behavior by the 4th cycle and peaked at approximately 45%. This is consistent with modeling studies also showing periodic behavior by the fourth cycle of the run (e.g., Figure 3). The CO peaks observed at the start of each regeneration step were the direct result of flushing off of the residual (unreacted) CO remaining at the end of the preceding adsorption step.

After the completion of the first testing campaign, several key design modifications were made to the MR-AR skid for enhanced performance and data collection. They included adding overtemperature thermocouples and additional flow control measures to the AR feed gas plumbing. “Quick to NDIR” ports were added for the MR section of the skid, secondary thermocouple logging equipment was either repaired or replaced, and the ARs were replumbed. The new thermocouples were connected to overtemperature switches, which were intended to shut off the syngas supply to the MR-AR skid if they read overtemperature or detected a faulty operation. This was in response to over-temperature conditions experienced, on one occasion, during the first testing campaign due to probable oxygen infiltration into the syngas from the gasifier. The primary modification to the MR-AR skid prior to the second testing campaign involved reducing the number of beds in series in each AR from 3 to 2 and reconfiguring the heater of the third bed as an in-line heater in between the two AR beds in series. This modification significantly improved the efficiency of the regeneration step. 

During the second testing campaign, MR, MR-AR experiments and CO_2_ breakthrough tests were carried out using both syngas and pure CO_2_ streams. The MR tests were carried out in a seven-tube membrane bundle running in a vertical orientation to avoid vibration-induced damage, which was discovered to be a problem in some instances during the first testing campaign when placing the membrane module in a horizontal position inside the oven. The membrane surface area for the bundle was 0.031 m^2^ and the reactor contained 635 g of the catalyst. Table 2 compares the experimental MR CO conversions with the multi-scale model predictions under the same operating conditions (the experimental data in the second and third rows were generated during the same run, but at different times of the day during which the syngas compositions from the two gasifiers were quite different from each other). A comparison of the measured and simulated MR CO conversions for the various datasets shows close agreement with the results. 

Several MR-AR cyclic tests were also carried out using the same vertical seven-tube bundle, with a surface area of 0.031 m^2^ and a catalyst loading of 635 g. Prior to each run, the AR beds were regenerated with steam and flushed out with N_2_ to remove any residual gases. For the reaction–adsorption run using syngas, only a portion (~40%) of the MR reject stream was fed to the AR. The AR A beds contained 451.8 g of the catalyst and 3505 g of the adsorbent, while the AR B beds contained 615 g of the catalyst and 3325 g of the adsorbent. 

**Figure 13:**
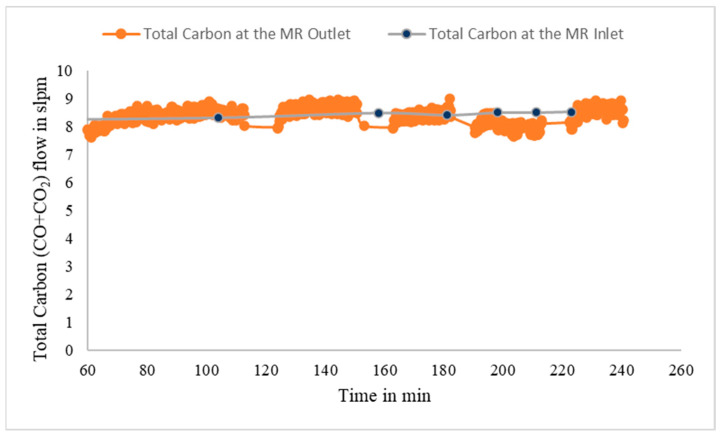
Total carbon (CO + CO_2_) flow in the MR inlet and MR reject-side exit.

Figure 13 shows the total carbon in the form of CO and CO_2_ fed to the MR and the corresponding total carbon detected at the MR reject-side exit once steady-state MR operation was obtained for one of these runs. For this run, the syngas composition remained fairly constant at (dry basis) H_2_: 28.37%, N_2_: 14.19%, CO: 26.12%, CO_2_: 30.95%, and H_2_S/CS_2_: 0.37%. After steady-state behavior was established, the average feed pressure, temperature, and flow rate (dry basis) were 230 psig (16.87 bar), 251 °C, and 9.633 slpm, respectively. The fact that, essentially, all the carbon in the syngas fed to the MR is recovered in the MR reject-side exit confirms that a negligible amount of carbon permeates through the membrane, thus ensuring high H_2_ purity for the MR permeate stream, and it is consistent with the high H_2_ permselectivity of the CMSM. 

**Figure 14:**
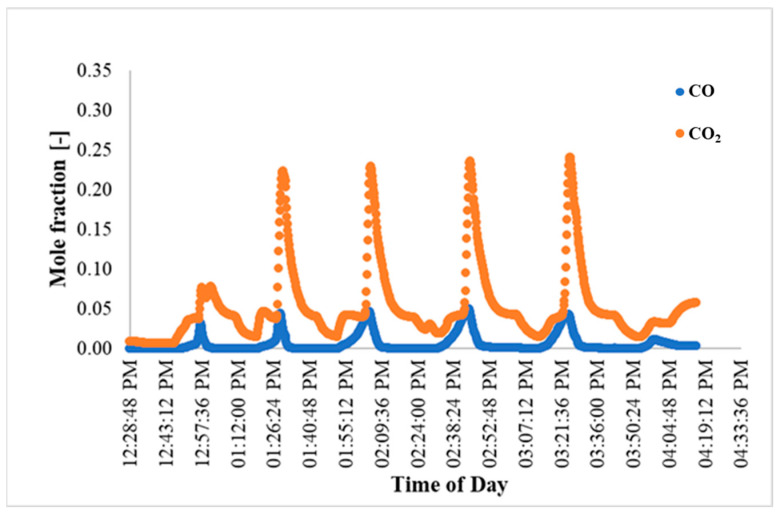
AR B CO and CO_2_ exit composition.

Figure 14 shows the performance of one of the ARs (AR B) during one of the cyclic runs. For this experiment, the syngas composition was fairly stable during the day of the experiment (dry basis, H_2_: 21%, N_2_: 35%, CO: 14%, CO_2_: 30%), the dry MR feed flow rate was ~23 slpm, the MR feed steam flow rate ranged between 9 and 11 slpm, and the feed pressure and temperature were 231 psig (16.93 bar) and 268 °C, respectively (at these conditions, the MR CO conversion was ~45%). Prior to each cyclic run, the beds were regenerated/flushed with N_2_/steam to remove any entrained gases. The AR A (AR B) beds contained a total catalyst mass of 451.8 g (615 g) and a total adsorbent mass of 3505 g (3325 g), as noted previously. Before the start of the cyclic runs, pure CO_2_ was fed at a predetermined pressure of ~10 psig (1.69 bar) to pre-saturate the beds, until the AR inlet and outlet CO_2_ concentrations were the same. The pure CO_2_ feed pressure was chosen to be approximately equal to the predicted (from the AR multi-scale model) CO_2_ partial pressure throughout the AR bed at the end of the AR regeneration phase when long-term AR behavior has been established. Pre-loading the bed with CO_2_ in this manner was intended to hasten the establishment of the long-term behavior in the AR, prior to the initiation of the cyclic testing. 

The AR feed pressure was 185 psig (13.76 bar) and the W_ads_/F_CO_2__ (ratio of the weight of adsorbent to the CO_2_ feed molar flow rate) was set at 4250 g/slpm, which meant that only a fraction of the reject stream of the MR was used as feed for the ARs. Each AR cycle run consisted of an adsorption phase of 20 min, followed by a regeneration phase of 20 min. Regeneration was carried out using a mixture of steam at a flow rate of ~37 slpm at 400 °C, and N_2_ at a flow rate of ~6 slpm. Figure 14 shows the CO/CO_2_ concentration data for AR-B for which steady cyclic behavior is established after four cycles, in agreement with the results of the multi-scale model.

### 2.3. Post-Mortem Materials Characterization

Upon completion of the second testing campaign, spent adsorbents and catalysts were tested for their activity and attrition characteristics. For the testing of catalyst activity, after the completion of the tests at CAER, the catalyst was removed from the MR vessel and tested for its activity in a lab-scale reactor at M&PT (during the transfer step, the catalyst was kept under a N_2_ atmosphere to avoid exposure to air since it is pyrophoric), employing simulated syngas. Prior to installing the catalyst inside the MR operating at CAER for the experimental testing to commence, the catalyst activity had been tested with simulated syngas in the same lab-scale reactor at the M&PT laboratories. The results of the spent catalyst indicated that its activity has remained quite stable and unaffected by the exposure to the gasifier syngas, which is in line with the findings of the laboratory study.

To enable catalyst and adsorbent pellet attrition testing, one of the AR vessels (Vessel 3, from AR A) was removed from the MR-AR skid to collect the used catalyst and adsorbent pellets to examine their attrition characteristics. At the time, the vessel was removed from the skid for catalyst and adsorbent pellet testing and it had undergone 38 adsorption/regeneration cycles and had been subjected to 165 h of cumulative syngas exposure. Visually, the used pellets appeared to be intact and similar in size and texture to the original pellets. For attrition testing, a modified version of the ASTM attrition method was used. While the rotating drum test is considered the standard method for testing catalysts, adsorbents, and other pelletized materials, the adsorbent/catalyst pellets being used in this project were exposed to other stressors: thermal/hydrothermal cycling and exposure to sulfur and heavy metal components. The attrition testing in the project, therefore, followed the ASTM method except for replacing the drum test with syngas exposure/cycling. The 850 μm screen was used as the determining cut-off for the attrition testing. The results of these tests indicated that the adsorbent shows the highest attrition rate (~0.16) with the catalyst showing a much smaller rate (~0.036), both meeting the attrition rate target of 0.2 set forth by DOE. Testing of the surface area for the used pellets showed no significant change from the unused adsorbent pellets.

### 2.4. Detailed TEA Study

Upon completion of the field-testing of the MR-AR process and based on the experimental performance of the field-scale system, a TEA study of the proposed MR-AR IGCC plant was carried out and compared to a reference IGCC plant with CCS (Case B5B of the reference document “Cost and Performance Baseline for Fossil Energy Plants—Volume 1: Bituminous Coal (IGCC) to Electricity. 24 September 2019”). The major differences between the MR-AR IGCC plant and the reference/baseline plant (Case B5B) are as follows:

- MRs and ARs replace the WGS reactors in the syngas clean-up (reaction) section of the plant. The MRs feature simultaneous H_2_ and CO_2_ generation and H_2_ removal, while the ARs feature simultaneous H_2_ and CO_2_ generation and CO_2_ removal.
- H_2_ and CO_2_ removal is facilitated by multidirectional steam flows during MR operation and AR regeneration, respectively.
- A single-stage Selexol unit is employed in the MR-AR IGCC plant for H_2_S removal only, as opposed to a dual-stage Selexol unit employed in the baseline case for both CO_2_ and H_2_S removal.
- Several of the MR-AR IGCC case studies presented feature elevated syngas humidification, which leads to the generation of humidified H_2_, whose combustion in the combustion turbine (CT) occurs at reduced temperatures and to the production of saleable N_2_ that is generated in the Air Separation Unit (ASU).
- A case study also involves ASU modification for the production and sale of both N_2_ and Ar.

The MR-AR section of the MR-AR IGCC plant was designed based on the simulation results of the experimentally validated multi-scale model, but apart from the modifications listed above, the other subsystems of the MR-AR IGCC plant are identical to those of the baseline plant, Case B5B, with little to no modification. In carrying out the detailed TEA, the following assumptions were utilized based on the reference plant:

- A capacity factor (CF) of 80% was used for the MR-AR IGCC plant.
- The combustion turbine (CT) operating philosophy is 2 × 232 MWe for a gross output of 464 MWe.
- Air pollution controls meet the applicable New Source Performance Standard (NSPS) targets for sulfur dioxide (SO_2_) [0.40 lb/MWh-gross], nitrogen oxides (NO_x_) [0.70 lb/MWh-gross], and particulate matter (PM) [0.07 lb/MWh-gross]. Mercury (Hg) and HCl removal devices meet the Utility Mercury and Air Toxics Standard (MATS) targets of [3 × 10^−6^ lb/MWh-gross] and [0.002 lb/MWh-gross], respectively. To meet these standards, H_2_S is converted into elemental sulfur in a Claus plant with tail gas recycle to limit SO_2_ emissions; NO_x_ is minimized with the use of low-NO_x_ burners (LNBs) and N_2_ dilution, as well as with syngas humidification; PM is controlled via water quench and the use of a syngas scrubber and a cyclone; Hg is controlled via sulfur-impregnated carbon beds; HCl is removed from the syngas scrubber with a brine concentrator and crystallizer.
- CO_2_ capture is greater than or equal to 90%.
- Steady-state process simulations with material and energy balances were used to size various process equipment for cost estimation.
- Capital and operating cost estimates are reported in 2018 dollars.
- The levelized price of coal (Illinois No. 6, Midwest) is $2.11/GJ on a higher heating value (HHV) basis and CO_2_ transport and storage (T&S) cost is $10/tonne ($9/ton).

Table S3 gives the overall performance of the MR-AR IGCC plant. The MR-AR IGCC power plant produces a net power output of 586 MW at a net plant efficiency of 35.5% (HHV basis). Table S4 gives the MR-AR plant levelized cost of electricity (LCOE) breakdown. The LCOE of the MR-AR plant, excluding CO_2_ T&S, is 130.7 $/MWh, compared to the baseline plant value of 144.2 $/MWh. In the MR-AR IGCC plant, steam is used to reduce the CT firing temperature; therefore, N_2_ is available as a saleable product. As a result, the N_2_ compression power requirement (36.58 MWe) can be eliminated. The elimination of the N_2_ compression power requirement results in a net power production of 623 MWe. The ASU produces 588 ton/h of 99.6% pure N_2_, of which 576 ton/h is available for sale at $30/ton. The remainder is used as a stripping medium in the AGR (single-stage Selexol) unit. If N_2_ sale is not possible (e.g., in the absence of a local market for N_2_—it is envisaged that the N_2_ market will grow largely due to its use in blue ammonia production, as demonstrated in Saudi Arabia and Japan), N_2_ can be used for CT firing temperature dilution, and in this case, N_2_ compression costs are reintroduced. The LCOE breakdown for N_2_ sales with/without N_2_ compression is shown in Table S5. If N_2_ is compressed but not sold, then the plant’s LCOE is the same as that shown in Table S4. 

An alternative is to incorporate another high-purity separation unit in the ASU that can deliver high-purity Ar. The process can then generate approximately 10,000 kg/h of Ar, which, at present, can be sold at a price of 4.0–6.0 $/kg. In this price range, the plant would generate an additional $280,320,000–$420,480,000 of revenue per year. However, modifying the ASU to separate high-purity Ar would incur increased capital (20%) and fixed operating (7%) costs. Increasing the capital cost of the ASU by 20% adds an additional $21,217,200 to the capital cost, which translates to $0.3659/MWh ($0.3441/MWh) for a net MR-AR power production of 586 MWe (623 MWe). If we assume the most extreme case, in which all the fixed operating costs are due to the ASU, then the maximum increase in the fixed operating cost is $7,412,800, which translates to $1.808/MWh ($1.701/MWh) for a net MR-AR power production of 586 MWe (623 MWe). The resulting COE for this case, which accounts for Ar sales at $4.0/kg, is shown in Table S6. The CO_2_ capture costs are shown in Table 3 for the various scenarios of the MR-AR IGCC plant.

Two critical technology parameters are affecting the COE in the TEA: the membrane lifespan and the sale price of N_2_ from the ASU. The membrane lifespan assumed for the TEA presented above is 10 years. A 5-year lifespan would increase the LCOE of the plant by 0.63% (0.60%), while a 2-year lifespan would increase the LCOE by 2.53% (2.40%), compared to a 10-year lifespan for 586 MWe (623 MWe) net power production. The modular nature of the MR process, which consists of several identical MR subunits, provides “economies of numbers” since the worst scenario of the 2-year membrane lifespan becomes less likely to occur in all MR subunits. The ASU produces 588 ton/h of pure N_2_, of which 576 ton/h could be sold at approximately $30/ton. However, the cost of semi-pure (99%) bulk N_2_ has been quoted at approximately $414/ton by different N_2_ providers such as Praxair and West Air Gas. This value accounts for transportation, storage, and delivery costs, however. If N_2_ was to be sold at $1/ton, this would mean the MR-AR design’s LCOE is 10.04% (15.32%) lower than the baseline LCOE for a net power production of 586 MWe (623 MWe). A N_2_ sale price of $414/ton would result in negative LCOE.

Therefore, in summary, when compared to the baseline IGCC plant (case B5B), the MR-AR plant with N_2_ sales at $30/ton (no N_2_ compression) achieves a higher carbon capture rate (96% vs. 90%), a higher net power production (623 MWe vs. 556 MWe), lower CO_2_ capture costs (39.9 $/tonne vs. 98.1 $/tonne), and lower LCOE (95.3 $/MWh vs. 144.2 $/MWh). When Ar is sold for $4.0/kg (no N_2_ compression scenario), the CO_2_ capture costs and LCOE are −4.6 $/tonne and 60.8 $/MWh, respectively. A summary of key performance indices for all cases is presented in Table 4. 

## 3. Conclusions

The overarching objective of this field-scale study was to field-validate the technical feasibility of a membrane- and adsorption-enhanced WGS reaction process that employed a CMSM-based MR followed by an AR for pre-combustion CO_2_ capture while demonstrating progress towards achievement of the overall performance goals of CO_2_ capture with 95% CO_2_ purity at a COE of 30% less than the baseline capture approaches. The main goal of this field-scale project was to advance the technology readiness level (TRL) of the proposed MR-AR transformative CO_2_ capture technology on its way to further pilot-scale testing. The project began at TRL 4, as the system prototype had already been validated in the laboratory on simulated syngas. The project ended at TRL 5, via scaling up of the prototype system and testing it on actual syngas at a host site (CAER at the UKy).

The project was carried out in two different phases. In Phase I, the team designed, constructed, and assembled the field-scale experimental MR-AR system, prepared the membranes, adsorbents, and catalysts, tested the field-scale unit with simulated syngas to validate functionality, and prepared a preliminary TEA of the technology. In Phase II, the team installed the unit at the test site at the UKy site and completed all utility connections and hook-ups, field-tested the novel MR-AR process in the field-scale system using real syngas, collected and analyzed experimental data, and completed a detailed TEA of the technology. A key aim of the project was to identify and address the technical and process risks and to generate information to advance the technology to the next stage of development.

All project milestones and success criteria were met. Specifically, (i) we prepared high-performance CMSM tubes that met the target H_2_ permeance (>1 m^3^/(m^2^.h.bar) or (>370.3 GPU)) and the target H_2_/CO selectivity of >80 at the relevant temperature (up to 300 °C) and pressure conditions (up to 25 bar) with a <10% decline in performance over each 250 h testing period; (ii) we prepared a sufficient quantity for the field testing of pelletized adsorbent for use in relevant conditions (250 °C < T < 450 °C, pressures of up to 25 bar) with a target adsorbent working capacity of >2.5wt.% and a target sorbent attrition rate of < 0.2; and (iii) updated the TEA of the process based on field-scale data to show that the proposed MR-AR IGCC technology met CO_2_ capture goals of 95% CO_2_ purity at a COE 30% less than baseline capture approaches. 

The CMSM, HTC adsorbents, and catalysts employed all exhibited very robust and stable performances during the long-term run (over a >250 h live syngas run). Furthermore, the proposed MR-AR IGCC system achieved a LCOE with a N_2_ sale price of $30/ton, with N_2_ compression (no N_2_ compression) of 101.2 $/MWh (95.3 $/MWh), which represents a 29.8% (33.9%) LCOE reduction in the baseline IGCC with carbon capture of 144.2 $/MWh. The proposed MR-AR IGCC delivers a CO_2_ capture cost of 44.8 $/tonne (39.9 $/tonne) vs. 98.1 $/tonne of the baseline capture case and a net power production of 586 MWe (623 MWe) vs. 556 MWe of the baseline capture case.

## Figures and Tables

**Table 1:** MR CO conversions, measured vs. simulated.

Dataset	Average Feed Composition [CO/CO_2_/H_2_/H_2_O/N_2_]	Feed Pressure [psig]	Temp. [°C]	Flow Rate [scfm]	Measured MR CO Conv. [%]	Simulated MR CO Conv.
12-9	23.99/36.25/21.13/7.57/11.06	285	250	0.482	53	46
12-13	16.35/38.75/16.57/25.84/2.49	249	240	0.585	39	41
12-14	12.75/32.76/13.90/30.10/10.49	255	249	0.614	51	52
12-15	14.58/29.90/14.37/29.87/11.28	245	263	0.626	52	46

**Table 2:** MR CO conversions, measured vs. simulated.

Dataset	Average Feed Composition [CO/CO_2_/H_2_/H_2_O/N_2_]	Feed Pressure [psig]	Temp. [°C]	Flow Rate [scfm]	Measured MR CO Conv. [%]	Simulated MR CO Conv.
1st MR Test	15.29/37.09/15.19/22.94/9.49	258	265	0.328	35	34
2nd MR Test	15.73/30.80/16.19/23.60/13.68	255	265	0.284	32	30
	9.34/24.12/9.05/37.37/20.12	220	265	0.284	75	74

**Table 3:** CO_2_ capture costs.

	Net Power	LCOE (Excluding T&S)	CO_2_ Captured	Cost of CO_2_ Captured
Plant	MW	$/MWh	tonne/MWh	$/tonne
Reference Non-capture Plant COE *	650	64.4	-	-
Baseline IGCC Plant COE (Case B5B)	556	144.2	0.814	98.06
MR-AR IGCC Plant (with N_2_ Compression)	585	130.7	0.823	80.60
MR-AR IGCC Plant with N_2_ Sales @ $30/ton (with N_2_ Compression)	586	101.2	0.823	44.76
MR-AR IGCC Plant with N_2_ Sales @ $30/ton (with no N_2_ Compression)	623	95.3	0.774	39.87
MR-AR IGCC Plant with Ar Sales @ $4.0/kg (with N_2_ Compression)	586	64.6	0.823	0.27
MR-AR IGCC Plant with Ar Sales @ $4.0/kg (with no N_2_ Compression)	623	60.8	0.774	−4.62

* The reference non-capture plant for the purpose of calculating the cost of CO_2_ captured is supercritical pulverized coal (SCPC) plant without capture (case B12A of reference document).

**Table 4:** Overall performance comparison of the baseline and MR-AR IGCC plant.

	Case B5B (Baseline)	MR-AR with N_2_ Compression	MR-AR (N_2_ Sale @ $30/ton, No N_2_ Compression)	MR-AR (N_2_ Sale @ $30/ton, N_2_ Compression)	MR-AR (Ar Sale @ $4.0/kg, No N_2_ Compression)	MR-AR (Ar Sale @ $4.0/kg, N_2_ Compression)	Target
Carbon Capture	90%	96%	96%	96%	96%	96%	N/A
CO_2_ Purity	99.5%	99.9%	99.9%	99.9%	99.9%	99.9%	95.0%
Net power Production (MWe)	556	586	623	586	623	586	N/A
LCOE (Excluding T&S), $/MWh	144.2	130.7	95.3	101.2	60.8	64.6	100.9
CO_2_ Captured Cost, $/tonne	98.1	84.7	41.3	46.5	−4.6	0.3	N/A

## Data Availability

The data presented in this study are available upon request from the corresponding author.

## Supplementary Materials

The following supporting information can be downloaded at: https://www.mdpi.com/article/10.3390/membranes14020051/s1, Figure S1: P&ID of the MR and the main oven. Figure S2: P&ID of the AR component. Table S1: AR design parameters and operating conditions (adsorption/regeneration). Table S2: AR adsorption test-run parameters. Table S3: MR-AR IGCC plant performance summary. Table S4: MR-AR plant LCOE breakdown. Table S5: MR-AR plant LCOE breakdown with N2 sale @ $30/ton, with/without N2 compression. Table S6: MR-AR plant LCOE breakdown with Ar sales @ $4.0/kg, with/without N2 compression, and a 20% and 7% increase in ASU capital cost and fixed operating cost, respectively.
