# Supplementary material for: Field-Scale Testing of a High-Efficiency Membrane Reactor (MR)—Adsorptive Reactor (AR) Process for H2 Generation and Pre-Combustion CO2 Capture
Source: Membranes (Basel). 2024 Feb 11;14(2):51. doi: 10.3390/membranes14020051 (PMC10890546; doi:10.3390/membranes14020051)
Supplement: Supplementary file 1 [file membranes-14-00051-s001.zip › membranes-2837314-supplementary.pdf]

Supplementary Materials

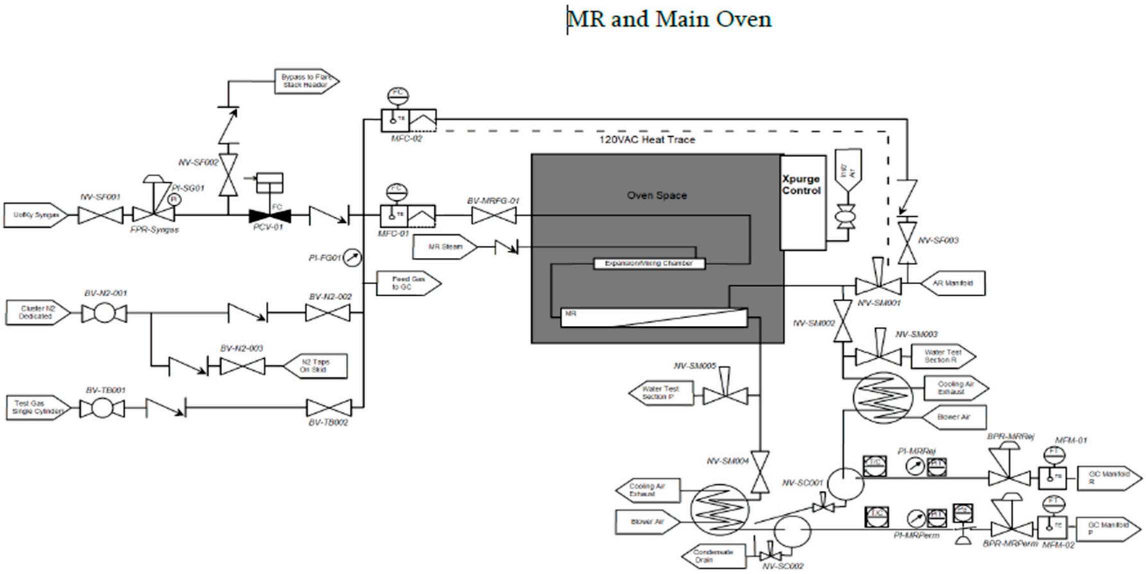

Figure S1: P&ID of the MR and the main oven.

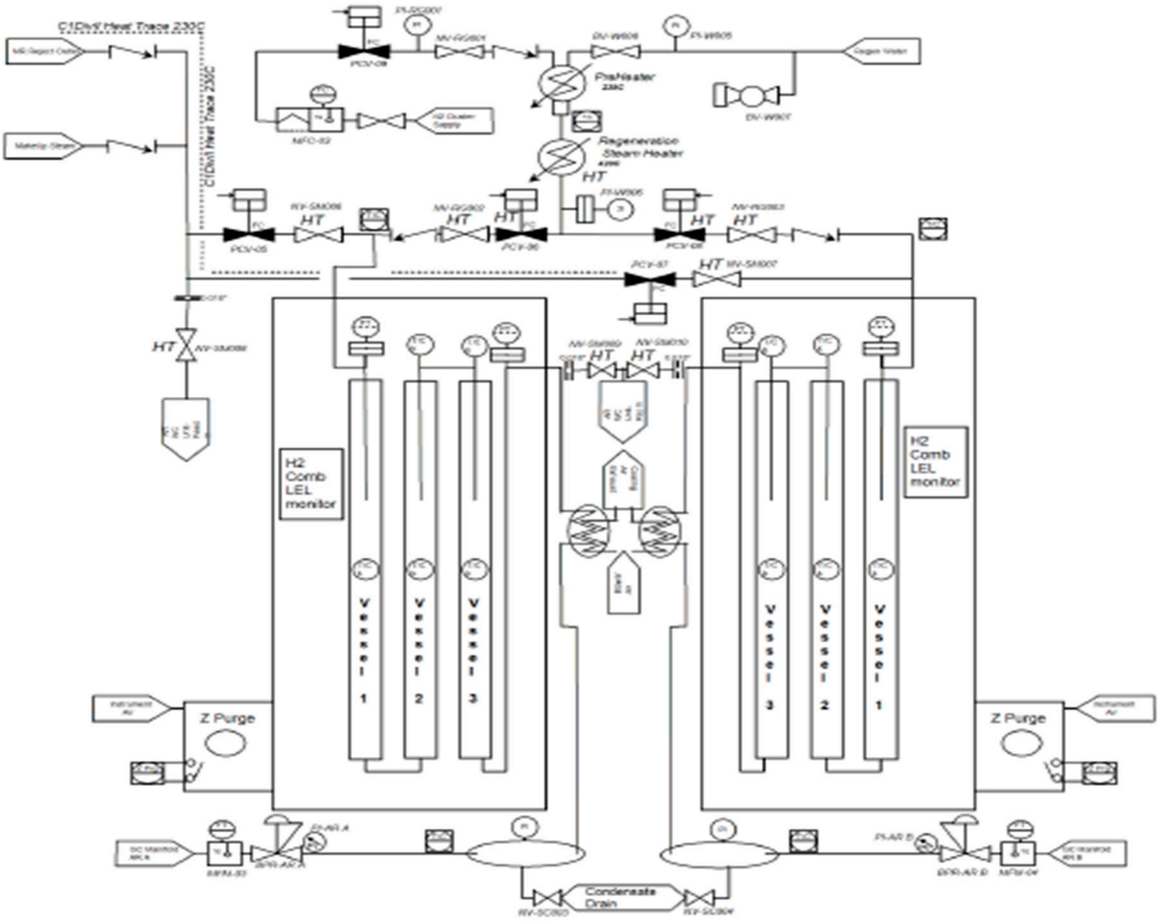

Figure S2: P&ID of the AR component.

Table S1: AR design parameters and operating conditions (adsorption/regeneration),

Length [′]	5
Diameter [″]	2.5
Heat Transfer Coefficient of Vessel [W/m²*K]	227
AR Vessel Wall Temperature [K]	523 (Ads.)/673 (Reg)
P [bar]	25
Feed T [K]	523 (Ads.)/673 (Reg)
Density of Catalyst [kg/m³]	592.68
Density of Adsorbent [kg/m³]	1,780
Catalyst Pellet Void Fraction [-]	0.35561
Adsorbent Pellet Void Fraction [-]	0.5
Mean Pore Diameter of Catalyst [m]	$6.3*10^{-9}$
Mean Pore Diameter of Adsorbent [m]	$6.3*10^{-7}$
Catalyst/Adsorbent Bed Void Fraction [-]	0.4717
**Feed (Regeneration Cycle)**	
100% steam	
Flow Rate [scfm]	2.5 - 10
**Feed (Adsorption Cycle)**	
**Composition (Molar Ratios)**	
CO_2/CO	1.2
H_2/CO	1.05
H_2O/CO	2
Flow rate [scfm]	2.5
**AR Fixed-Bed Composition**	
W_{ads} [kg]	2.72
W_{cat} [kg]	1.75
W_{ads}/W_{cat}	1.55
Pellet Diameter [″]	0.25

Table S2: AR adsorption test-run parameters.

	Value	Unit
**AR Test Feed**		
Pressure (Avg.)	16.35	bar
Temperature (Avg.)	517	K
Feed Mole Fraction: CO_2 N_2	0.3, 0.7	-

Flow Rate (Avg.)	0.326	scfm
**AR Configuration**		
AR Vessel Length (3 AR in Series, Each 5 ft long)	15	ft
AR Vessel Diameter	2.5	in
Packing Density (Catalyst)	568	kg/m^3
Packing Density (Adsorbent)	1700	kg/m^3
Adsorbent Pellet Pore Diameter	23.07	nm
Max. Adsorbent Loading/Train (3 AR in Series)	1639	g
Wads/Wcat	8	-

Table S3: MR-AR IGCC plant performance summary**.**

**Performance Summary**		
	**Case B5B**	**MR-AR**
Combustion Turbine Power, MWe	464	464
Sweet Gas Expander, MWe	3	3
Steam Turbine Power, MWe	274	274
**Total Gross Power, MWe**	**741**	**741**
Air Separation Unit Main Air Compressor, kWe	71,280	71,280
Air Separation Unit Booster Compressor, kWe	5,610	5,610
Nitrogen Compressors, kWe	36,580	36,580
CO_2 Compression, kWe	31,670	9,327
Acid Gas Removal, kWe	11,550	2,950
Balance of Plant, kWe	28,080	28,977
**Total Auxiliaries, MWe**	**185**	**155**
**Net Power, MWe**	**556**	**586**
HHV Net Plant Efficiency (%)	33.7%	35.5%
HHV Net Plant Heat Rate, kJ/kWh (Btu/kWh)	10,675 (10,118)	10,131 (9,603)
HHV Cold Gas Efficiency, %	79.0%	79.0%
HHV Combustion Turbine Efficiency, %	36.4%	36.4%
LHV Net Plant Efficiency (%)	35.0%	36.8%
LHV Net Plant Heat Rate, kJ/kWh (Btu/kWh)	10,296 (9,759)	9,772 (9,262)
LHV Cold Gas Efficiency, %	75.7%	75.7%
LHV Combustion Turbine Efficiency, %	42.8%	42.8%
Steam Turbine Cycle Efficiency, %	43.1%	43.1%
Steam Turbine Heat Rate, kJ/kWh (Btu/kWh)	8,356 (7,920)	8,356 (7,920)

Condenser Duty, GJ/hr (MMBtu/hr)	1,555 (1,474)	1,555 (1,474)
AGR Cooling Duty, GJ/hr (MMBtu/hr)	147 (140)	147 (140)
As-Received Coal Feed, kg/hr (lb/hr)	218,895 (482,580)	218,895 (482,580)
HHV Thermal Input, kWt	1,649,926	1,649,926
LHV Thermal Input, kWt	1,591,374	1,591,374
Raw Water Withdrawal, $(m^3/min)/MW_{net}$ (gpm/MW_{net})	0.037 (9.9)	0.034 (9.0)
Raw Water Consumption, $(m^3/min)/MW_{net}$ (gpm/MW_{net})	0.030 (7.9)	0.026 (6.9)
O_2:As-Received Coal	0.760	0.760

Table S4: MR-AR plant LCOE breakdown.

	**MR-AR**		**Baseline**	
**Net Power Production (MWe)**	**586**		**556**	
**Component**	**Value, $/MWh**	**Percentage**	**Value, $/MWh**	**Percentage**
Capital	64.1	46%	75.2	49%
Fixed	25.8	19%	27.2	18%
Variable	19.4	14%	19.3	13%
Fuel	21.4	15%	22.5	15%
**Total (Excluding T&S)**	**130.7**	**N/A**	**144.2**	**N/A**
CO_2 T&S	8.2	6%	8.1	5%
**Total (Including T&S)**	**138.9**	**N/A**	**152.3**	**N/A**

Table S5: MR-AR plant LCOE breakdown with N_2 sale @ $30/ton, with/without N_2 compression.

	**MR-AR**		**Baseline**	
**With N_2 Compression**				
**Net Power Production (MWe)**	**586**		**556**	
**Component**	**Value, $/MWh**	**Percentage**	**Value, $/MWh**	**Percentage**
Capital	64.1	59%	75.2	49%
Fixed	25.8	24%	27.2	18%
Variable	19.4	18%	19.3	13%
Fuel	21.4	20%	22.5	15%
N_2 sales @ $30/ton (with N_2 Compression)	-29.5	-27%	N/A	N/A
**Total (Excluding T&S) [with N_2 Compression]**	**101.2**	**N/A**	**144.2**	**N/A**

CO_2 T&S	8.2	8%	8.1	5%
**Total (Including T&S) [with N_2 Compression]**	**109.5**	**N/A**	**152.3**	**N/A**
**Without N_2 compression**				
**Net Power Production (MWe)**	**623**		**N/A**	
Capital	60.4	59%	N/A	N/A
Fixed	24.3	24%	N/A	N/A
Variable	18.3	18%	N/A	N/A
Fuel	20.1	20%	N/A	N/A
N_2 sales @ $30/ton (no N_2 Compression)	-27.8	-27%	N/A	N/A
**Total (Excluding T&S) [no N_2 Compression]**	**95.3**	**N/A**	**N/A**	**N/A**
CO_2 T&S	7.7	8%	N/A	N/A
**Total (Including T&S) [no N_2 Compression]**	**103.0**	**N/A**	**N/A**	**N/A**

Table S6: MR-AR plant LCOE breakdown with Ar sales @ $4.0/kg, with/without N_2 compression, and a 20% and 7% increase in ASU capital cost and fixed operating cost, respectively.

	**MR-AR**		**Baseline**	
**With N_2 Compression**				
**Net Power Production (MWe)**	**586**		**556**	
**Component**	**Value, $/MWh**	**Percentage**	**Value, $/MWh**	**Percentage**
Capital	64.5	89%	75.2	49%
Fixed	27.6	38%	27.2	18%
Variable	19.4	27%	19.3	13%
Fuel	21.4	29%	22.5	15%
Ar sales @ $4.0/kg (with N_2 Compression)	-68.3	-94%	N/A	N/A
**Total (Excluding T&S) [with N_2 Compression]**	**64.6**	**N/A**	**144.2**	**N/A**
CO_2 T&S	8.2	11%	8.1	5%
**Total (Including T&S) [with N_2 Compression]**	**72.9**	**N/A**	**152.3**	**N/A**
**Without N_2 Compression**				
**Net Power Production (MWe)**	**623**		**N/A**	
Capital	60.7	89%	N/A	N/A
Fixed	26.0	38%	N/A	N/A
Variable	18.3	27%	N/A	N/A

Fuel	20.1	29%	N/A	N/A
Ar sales @ $4.0/kg (no N_2 Compression)	-64.2	-94%	N/A	N/A
**Total (Excluding T&S) [no N_2 Compression]**	**60.8**	**N/A**	**N/A**	**N/A**
CO_2 T&S	7.7	11%	N/A	N/A
**Total (Including T&S) [no N_2 Compression]**	**68.6**	**N/A**	**N/A**	**N/A**
